# Protocol- Comprehensive Care Package to reduce deaths among adult persons diagnosed with Tuberculosis in Kerala, India (CCp-K)-An implementation project

**DOI:** 10.1016/j.mex.2025.103572

**Published:** 2025-08-19

**Authors:** Raman Swathy Vaman, Kalaiselvi Selvaraj, Dina Nair, Abey Sushan, Aparna Mohan, Krishna Devadhas Sulochana, Jeen Melfha, Arun Paramarthalingom Vijayalekshmi, Vyas Sukumaran, Naveen Anaswara, Sujith Sukumaran, Annadurai Jeyakumar, Rakesh Purushothama Bhat Susheela, Rajaram Kizhakkekandiyil, Hemant Deepak Shewade

**Affiliations:** a Department of Health and Family Welfare, Government of Kerala, India; b All India Institute of Medical Sciences, Madurai, Tamil Nadu, India; c ICMR- National Institute for Research in Tuberculosis, Chennai, Tamil Nadu, India; d WHO NTEP division, Kerala, India; e ICMR- National Institute of Epidemiology, Chennai, Tamil Nadu, India; f The Union, New Delhi, India

**Keywords:** Tuberculosis, Implementation research, Operational research, India, Differentiated TB care, TB mortality

## Abstract

This implementation project aims to reduce TB mortality by at least 25% through early triaging and integrated management of co-morbidities in Kerala, India. The package will be implemented across Kerala’s public health facilities, targeting all adult TB patients for systematic triaging for severe illness, uncontrolled diabetes mellitus, alcohol dependence, and tobacco dependence. Triage-positive individuals will be referred for comprehensive assessment and inpatient care to nodal treatment centres. This research will follow a mixed-methods approach with quantitative components assessing feasibility, burden, and programmatic outcomes, and qualitative interviews exploring facilitators and barriers. Triaging will be integrated into routine workflows with healthcare worker training and system monitoring using customized indicators. If found feasible and effective in improving programmatic outcomes such as early triaging and inpatient referral, this model offers potential for scale-up across India to reduce preventable TB deaths through differentiated care and targeted comorbidity management.

• Implements a comprehensive care package to reduce TB deaths through early triaging and comorbidity management

• Leverages existing public health infrastructure to deliver enhanced TB care within routine programme settings

## Graphical abstract

**Figure:**
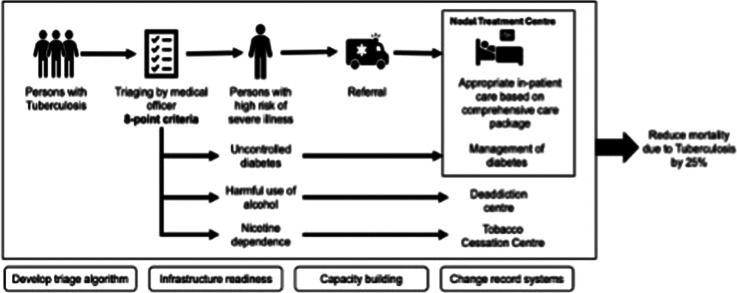

## Specifications table

**Table:** 

Subject area	Medicine and Dentistry
More specific subject area	Public Health
Name of your protocol	Protocol- Comprehensive Care Package to reduce deaths among adult persons diagnosed with Tuberculosis in Kerala, India (CCp-K)- An implementation project
Reagents/tools	Not applicable
Experimental design	Not applicable
Trial registration	Not applicable
Ethics	This study will be performed in line with the principles of the Declaration of Helsinki. The study is approved by the Institutional Human Ethics Committee of ICMR-NIE Chennai, Approval no NIE/IHEC/A/202408-06 dated 18/09/2024. Necessary administrative approvals from the Government of Kerala for implementing the project will be taken. For the quantitative component of the study, a verbal consent /implied consent to mimic the implementation in routine programme setting and enable assessment of feasibility as approved by the ethics committee will be used. A written informed consent from the participants will be obtained before initiating the qualitative interviews. All data will be coded and anonymised before analysis.
Value of the Protocol	• This protocol offers a practical framework for integrating early triage and management of severe illness, diabetes mellitus, alcohol dependence and tobacco dependence into routine TB care—applicable to low- and middle-income countries facing similar health system challenges. • By leveraging existing infrastructure and implementing within the National TB programme, the protocol will produce operational evidence on feasibility, acceptability, and programmatic outcomes of the framework—critical for global policy adaptation and health systems strengthening. • The protocol directly contributes to the global agenda of reducing TB deaths through timely inpatient care and risk-targeted interventions, aligning with WHO End TB targets and offering lessons for differentiated TB care in diverse epidemiological contexts.

## Background

Globally an estimated 10.8 million people fell ill with Tuberculosis (TB) in 2023 with South-East Asia region contributing nearly half of it [1]. In India there was an estimated 2.8 million cases and 323200 deaths due to TB in 2023 [1]. Post Covid-19 pandemic, the country is moving towards accelerated efforts to attain the End TB targets by 2025.

Kerala, a southern state in India with a population of approximately 33 million, is known for its robust public health system and high human development indicators. The state has consistently achieved strong performance in maternal and child health, immunization coverage, and health service access. In 2023, Kerala reported a 2.5 times lower TB notification than the national average [2]. As a pioneer in implementing various health programmes successfully, the state had launched the ambitious “Kerala TB elimination mission”- a four year strategic plan in 2017 [3]. Though improvements had been made on active case finding and management, still the case fatality among persons with TB (PwTB) in Kerala is more than twice the national average [2]. This is further complicated by the high prevalence of comorbidities such as hypertension (44%), diabetes mellitus (24%) and pre-diabetes (18%) in Kerala [4].

Malnutrition, comorbidities, alcohol and substance abuse were proven to be risk factors for death among PwTB [5]. A recent Global Burden of Disease study determined that dietary risks, high fasting plasma glucose, low physical activity, tobacco, and alcohol use were the main contributors to TB age-standardized Disability-Adjusted Life Years and mortality [6].

Differentiated TB care refers to a patient-centered approach that stratifies individuals with TB based on their clinical severity, comorbid conditions, and social vulnerabilities, and delivers tailored care packages accordingly. Rather than offering a uniform treatment pathway, this model emphasizes early identification of high-risk individuals such as those with severe undernutrition, respiratory insufficiency, or uncontrolled diabetes and links them to more intensive clinical management, including inpatient care [7]. Differentiated TB care including early triaging of severely ill TB patients, effective chemotherapy and comorbidity management are crucial in reducing TB mortality [7,8]. Previously many LMICs have integrated TB care with non-communicable diseases at various levels and identified lack of programmatic budget, and additional stress on providers due to increased workload as major barriers to implementation [9]. India’s national TB elimination programme (NTEP) in January 2021, recommended severity assessment for all notified patients at diagnosis and referral of severely ill TB patients for inpatient care [10]. The NTEP severity assessment tool used a comprehensive 16 item checklist for screening and has not been implemented uniformly across the country due to operational issues. As most of the TB deaths are early deaths (within two months) and half of early deaths happen within the first two weeks of diagnosis, this mandates a quick triaging for severe illness at the time of diagnosis [[11], [12], [13]].

Currently the patients notified under National Tuberculosis Elimination Programme (NTEP) is screened for presence or absence of Diabetes Mellitus (DM), alcohol use, tobacco use and no systematic triage is being conducted [14]. A structured follow-up mechanism is also lacking in this system. One of the evidence-based implementation project of differentiated TB care model from the neighbouring state (TN-KET) focus on early triaging for severely ill TB patients using a five point triage tool [15,16]. This project has successfully demonstrated the feasibility of early triage and differentiated care in programmatic settings in TamilNadu [[16], [17], [18], [19]]. However, considering the increased burden on non-communicable diseases and risk factors, the differentiated TB care model need to cater for these co-morbidities. In response to this high TB mortality in Kerala and the need for integrated risk-based care, we co-developed a Comprehensive Care Package (CCp-K) in collaboration with the state health system. This package targets all adult TB patients diagnosed at public health facilities and includes systematic triaging for severe illness, screening for uncontrolled diabetes mellitus, and assessment of alcohol and tobacco dependence, with structured referral and inpatient care pathways. The current study aims to implement this package across Kerala’s public health system and evaluate its feasibility, programmatic outcomes, and acceptability through a mixed-methods implementation research design. This package will be implemented across Kerala health system under the NTEP with technical support from Indian Council of Medical Research-National Institute of Epidemiology, Chennai, All India Institute of Medical Sciences, Madurai and Indian Council of Medical Research-National Institute for Research in Tuberculosis, Chennai.

## Description of protocol

### Aim

We aim to implement systematic triaging for all adults (≥18 years) with Tuberculosis diagnosed from all public health facilities for severe illness, uncontrolled diabetes mellitus, alcohol dependence and tobacco dependence followed by referral, comprehensive clinical assessment and inpatient care in Kerala from August 2025. As a result, we expect to reduce the deaths among diagnosed TB patients relatively by at least 25% by 2027.

### Specific objectives

1. To determine the feasibility (coverage, timeliness and completion) of implementing a comprehensive care package (CCp-K) in public health facilities
2. To estimate the proportion of patients triaged and referred for comprehensive clinical assessment and inpatient care based on severe disease and severe comorbidities
3. To determine the patient and health system factors associated with not getting triaged and not getting admitted for these conditions
4. To compare the effect of the comprehensive care package in reducing TB deaths (early and overall) between August 2024 and December 2025
5. To explore the perceived feasibility and relevance, facilitator and barriers for implementing CCp-K in public health facilities
6. To document the implementation process of adopting CCp-K, acceptability and integrating these components of care under NTEP from healthcare providers (involved in triage and referral linkage) perspectives

## Methods

### Study setting

This implementation project will be conducted in routine public health system settings of Kerala, India led by the State TB cell utilizing existing resources. The investigators will provide technical support in planning, implementation and monitoring along with World Health Organization (WHO) India.

Kerala, the southernmost state of India has a population of ≈ 33 million. The state had a presumptive TB case examination of 1200 per 100,000 population in 2023 [2]. The TB notification rate was 63 per 100,000 population (national average 153). In Kerala, 33% of tested TB patients were diagnosed with diabetes mellitus, 13% had tobacco usage and 13% had alcohol use among those screened [2]. The overall estimated prevalence of diabetes mellitus in Kerala is 25.5%, alcohol use is 12.4% and tobacco usage is 17% in Kerala [4,20,21]. Among the cohort of patients started treatment in 2020 in Kerala, 16770 (81%) had treatment success and 1668 (8.1%) died [2].

The state NTEP infrastructure includes 14 NTEP districts. Peripheral health institution (PHI) within the NTEP includes facilities that notify TB patients. The public PHIs have at least one medical doctor and laboratory access either through a TB diagnostic centre (TDC) or a sample transportation linkage to the nearest TDC. Ni-kshay application (a case-based, web-based electronic TB information management system) is the primary information management system (https://www.nikshay.in). Along with that paper-based registers are maintained at each of these PHI and has a line-list of patients notified, their management, and the treatment outcomes. Each sub-district level unit (TB unit) has a senior TB treatment supervisor (STS) and a Senior Treatment Laboratory Supervisor (STLS) & Medical Officer for TB Control (MOTC). The programme in each district is supervised by the District TB Officer (DTO) and the programme management unit at the District TB centre (DTC). Every district has a dedicated pulmonologist at the DTC. There are other dedicated key staff to support the STS, called TB-health visitor in the urban areas and drug-resistant TB coordinator to monitor DR-TB patients. Patients receive daily treatment under direct observation of a health care provider, community volunteer or a family member. For training the Health care worker (HCW) we will utilize the existing training team present in each district. Currently patients with TB and DM are managed according to the guidelines for joint NCD-TB collaborative guidelines by the CTD. There are at least one functional tobacco cessation clinics and deaddiction centres (“Vimukthi” centers (meaning *“liberation”* in Malayalam) one in each district) at district headquarters. Currently, before initiation of treatment for TB weight and substance use status is documented. However, these findings are not used to classify the severity of TB or referral for inpatient care. Similarly, substance use status is documented in the web portal and not used for referral to higher facilities.

### Implementation phase

The implementation phase will be preceded by a two month preparatory phase (June-July 2025), getting administrative approvals; developing standard operating procedures for triaging, referral, comprehensive and inpatient care; identifying the nodal inpatient care facilities within the districts and TB beds along with the nodal physicians in these facilities, development of tools for recording reporting and monitoring framework; training of NTEP staff, nodal physicians, ensuring that all have access to relevant tools and pilot the CCp-K. We will be introducing a comprehensive care package as described below in routine setting utilizing existing resources by the health system and NTEP programme.

**The comprehensive care package (CCp-K)**

The CCp-K includes four components.

1. Triaging all diagnosed patients with TB using the triage tool (Box 1) and referral to nodal treatment centre for assessment by physician/ pulmonologist and in patient management.

2. Screening of all patients with TB and diabetes for fasting blood glucose (FBG) at diagnosis, and 2 months and referring for insulin and inpatient care if FBG ≥250/ mg/dl or HbA1C >10% [22]

3. Assessing all patients with alcohol use for harmful use of alcohol using “CAGE questionnaire” [23] and referring to deaddiction centres (“Vimukthi”) for management.

4. Assessing all patients with smoking for nicotine dependence using “Heaviness of Smoking Index” [24] adapted from the Fagerstrom test for Nicotine dependence and referring to Tobacco cessation centres for moderate and high addiction.

Box 1 **Triage tool for severe illness at diagnosis among adults (≥15 years) with TB notified form public facilities**

If at least one of the following is present, then the person with TB is ‘high risk of severe illness / triage-positive’ (requires referral for comprehensive assessment, confirmation of severe illness and inpatient care)

1. Body mass index (BMI) less than or equal to (≤) 14.0 kg/m^2 #^

2. BMI less than or equal to (≤) 16.0 kg/m^2^ with leg swelling ^#^

3. Respiratory rate more than (>) 24 per minute ^##^

4. Oxygen saturation less than (<) 94% ^##^

5. Not able to stand without support (poor performance status - standing with support/squatting/sitting/bedridden)

TB-tuberculosis; NTEP – national TB elimination programme

*Reprinted from Shewade HD et al [25] under a CC BY license, with permission from MDPI, Copyright MDPI 2021, tool adapted from Bhargava A et al [6]; # very severe undernutrition indicators; ## respiratory insufficiency indicators

Alt-text: Unlabelled box

**Fig. 1:**
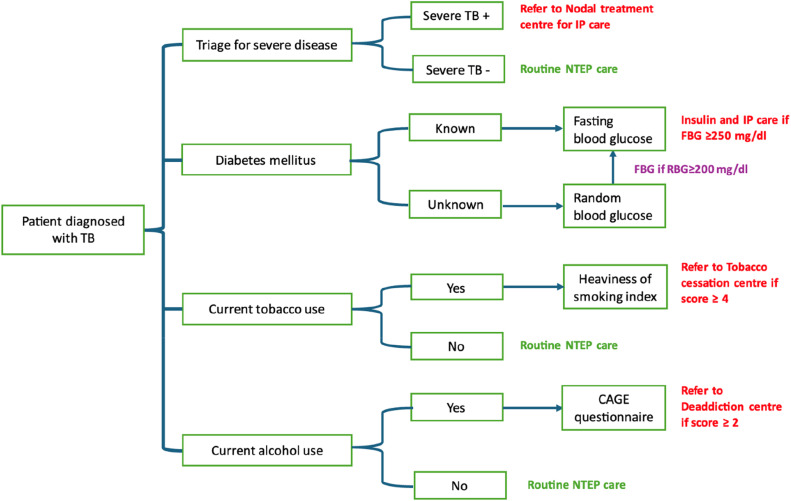
The CCp-K care cascade.

**The Care Cascade** (Fig. 1)

### Triaging

We will introduce a paper-based triage tool (to be used at TB diagnosis) in all the public PHIs (Annexure 1) integrated with the treatment documentation book. Triaging will be done by the Medical officer or staff nurse (in MOs absence only) of the facility where the patient is evaluated and diagnosed with TB. If any one of the five-point criteria is positive, it implies that the patient is at high risk of severe illness and should be referred to the nodal treatment centre. We will adapt the standard operating procedures from the TN-KET project available in public domain for using the triage tool [25].

### Referral and appropriate care

For triage-positive adults, the concerned STS will facilitate the referral (using public ambulance service) with the support of MO-PHI & MOTC to nodal inpatient care facilities for comprehensive clinical assessment (using paper-based case record form), confirmation of severe illness and inpatient care. Box 2 contains the criteria for confirmation of severe illness after comprehensive assessment. The National technical guidelines for comprehensive package for differentiated care for TB patients will be utilized for management of inpatients [10].

### Comorbidity management

For patients with diabetes mellitus, we will screen them for FBG on the immediate next day and if FBG is more than 250 mg/dl or HbA1C >10% the patient will be referred to nodal centre for insulin and inpatient care. Those patients who are current smokers will be assessed using the two questions of “Heaviness of smoking index” and the patient will be refereed to tobacco cessation centres if the score is ≥ 4 indicating moderate to high dependence. Patient who currently use alcohol will be assessed for alcohol dependence using CAGE criteria and referred to deaddiction centres if the score is ≥ 2.

Box 2 Criteria for patients requiring inpatient care* among adults (≥15 years) with TB based on CTD 2021 technical guidance on differentiated care of TB patients in India [10].

**Table:** 

Criteria		Considered to be emergency**	Scoring criteria	Score assigned
1	Pulse rate (per minute)		<60 >100 (persistent after 30 minutes)	2 2
2	BMI (kg/m^2^)	Yes (<14)	<14 <16 with pedal oedema >40	1 1 1
3	MUAC (cm)		<16	1
4	Temperature (Celsius)	Yes (<35, >41)	<35 >41	2 2
5	Blood pressure (mm Hg)	Yes	Hypertension (≥140/90) Hypotension (diastolic below 60)	2 2
6	Respiratory rate (per minute)	Yes (>24)	18-24 25-30 >30	1 2 3
7	Oxygen saturation (%)	Yes (<94)	90-93 85-90 <85	1 2 3
8	Hemoglobin (g%)	Yes (<7)	<7	2
9	Icterus		Present	1
10	Pedal Oedema		Present	1
11	General condition	Yes (if unable to walk, drowsy, unconscious)	Inability to walk but conscious and oriented Conscious, not oriented Drowsy	1 2 3
12	HIV		Positive and on ART Positive and not on ART	1 2
13	Random blood sugar		<70 >200	2 2
14	Total white blood cell count		TC > 11000 TC < 4000	1 1
15	Chest radiograph	Yes (massive pneumothorax, hydropneumothorax)	Hydropneumothorax Bilateral consolidation	3 2
16	Hemoptysis	Yes	Present	3

TB - tuberculosis, CTD – central TB division, BMI – body mass index, MUAC – mid upper arm circumference, HIV – human immunodeficiency virus, ART – antiretroviral treatment

*Total score more than one – to be provided inpatient care, total score more than three – to be provided inpatient care in a facility with intensive care unit. **If there is an indicator suggesting emergency, the inpatient care should be provided irrespective of the total score.

*Modifications to the criteria provided by CTD 2021 technical guidance: blood sugar of >128 replaced with >200. In addition, inpatient care may be provided irrespective of the total score or presence / absence of emergency criteria if the treating physician feels the need for inpatient care.

Alt-text: Unlabelled box

### Implementation strategies

The implementation strategies proposed for the CCp-K project were systematically classified using the Expert Recommendations for Implementing Change (ERIC) framework of discrete implementation strategies [26]. The strategies are detailed below:

**1. Involve Executive Boards**

A stakeholder mapping exercise will be conducted to identify key individuals and institutions relevant to the implementation of the CCp-K project. The Mendelow’s Power-Interest Matrix will be employed to classify stakeholders based on their level of influence and interest in the project [27]. Stakeholders with high power and/or high interest will be approached to orient the scope of the project. A series of consultative meetings are scheduled to sensitize and engage these stakeholders including state and district-level officials in the differentiated TB care model. These meetings are aimed at building consensus, clarifying roles, inter-program coordination and contextualizing the implementation strategy. Feedback from these stakeholders will also guide the adaptation of tools, referral pathways, and capacity-building plans before field implementation.

**2. Assess for Readiness and Identify Barriers and Facilitators**

To evaluate the feasibility of the proposed model, in-depth interviews will be conducted with stakeholders at both the state and district levels. The interviews will explore perceptions of the relevance of the CCp-K project, as well as potential barriers and facilitators to its implementation, aligned with the Consolidated Framework for Implementation Research (CFIR) [28].

**3. Conduct Local Consensus Discussions**

A prototype model, developed through internal consultations among public health experts and practitioners involved in national TB programs, will be presented to key stakeholders including WHO consultants. These discussions will aim to establish a consensus on the operational design. Based on feedback from these sessions, the model will be refined and finalized prior to field implementation.

**4. Develop Resource Materials and Conduct Ongoing In-Service Training**

Training resource materials such as PowerPoint presentations, guidelines, and standard operating procedures (SOPs) will be developed by the CCp-K team through a participatory approach. A district-level training-of-trainers (ToT) program will be conducted in the pre-implementation phase, following which the trained personnel will cascade the training to healthcare workers (HCWs) in their respective districts. A training calendar will guide these activities to ensure systematic roll-out.

**5. Develop a Triage Algorithm and Referral Flow**

A standardized triage algorithm will be developed to screen all adult public-sector notified TB patients at the time of diagnosis for (a) severe illness, (b) diabetes mellitus (DM), (c) alcohol dependence, and (d) nicotine dependence. Patients identified as high-risk or requiring further intervention will be referred through a structured referral flow to designated nodal treatment centers (NTCs), deaddiction services (“Vimukthi” centers), and tobacco cessation clinics as appropriate. The algorithm and referral flow will be field-tested and validated in consultation with clinical experts and incorporated into the operational workflow of healthcare facilities in the district.

**6. Change Physical Structure**

Following consultations with the State TB Officer (STO) and District TB Officers (DTOs), a government order will be issued designating Nodal Treatment Centres (NTCs) in each district. Each NTC will have dedicated inpatient beds for managing triaged patients with severe illness. PHIs will be mapped to their respective NTCs. Infection prevention and control measures, including airborne precautions, will be integrated into the infrastructure and workflow of the NTCs.

**7. Logistics and Supply Chain Management**

Essential supplies including drugs (e.g., insulin), equipment, and consumables will be provisioned at PHIs, NTCs, and deaddiction centres. Essential supplies will be met through the existing health system supply chains under NTEP, NCD, COPD and deaddiction programmes. The research team will coordinate with programme managers and procurement agencies to ensure alignment without direct project-led procurement. We will document incremental resource needs and cost differentials (e.g., additional diagnostics, days of inpatient care, consumables) required to implement the CCp-K package. These insights will be shared with state programme managers and policymakers to support potential budgetary planning and policy integration. We will facilitate coordination with Kerala Medical Services Corporation Ltd. (KMSCL) and respective programme divisions to ensure real-time alignment of supply with demand. Specific attention will be given to ensure availability of materials for infection control, including disinfectants and sterilization agents.

**8. Change Record Systems**

As the current Ni-kshay portal does not capture co-morbidity data comprehensively, a monthly supplementary reporting format will be introduced at the PHI level. This report will include data on identified comorbidities and referral actions taken for each notified TB patient, until such time the official system is updated to incorporate these parameters (Table 1).

**Table 1:** Reporting format for CCp-K at the PHI level.

Conditions (col 1)	Existing comorbidity (col 2)	Triaged (col 3)	Triage positive (col 4)	Referred (col 5)	Coverage (col 6)
Severe illness	-				Assessed for 5 point/N
DM	k/c/o DM				Assessed for /col 2
Alcohol use	Use of alcohol				Assessed for /col 2
Tobacco use	Use of tobacco				Assessed for /col 2

**9. Provide Supervision**

Implementation progress will be tracked through the existing National TB Elimination Programme (NTEP) supervisory mechanisms. A complementary monitoring and evaluation (M&E) system will be established using hybrid metrics based on the RE-AIM (Reach, Effectiveness, Adoption, Implementation, Maintenance) and PRISM (Practical, Robust Implementation and Sustainability Model) frameworks [29,30]. Performance indicators for each district will include Proportion of adult TB patients screened for severe illness and comorbidities at diagnosis (target: ≥80%), proportion of triage-positive patients receiving comprehensive assessment (target: ≥80%), admission rate among those confirmed with severe illness (target: ≥80%), median duration of inpatient care (target: 7 days), proportion of persons with uncontrolled DM admitted for insulin initiation (target: ≥80%), proportion of persons with alcohol dependence referred to deaddiction services (target: ≥80%), proportion of persons with nicotine dependence referred to tobacco cessation centers (target: ≥80%), persons with nicotine dependence referred to tobacco cessation center - 80%. An 80% threshold was selected based on programmatic experience from the TN-KET project and operational discussions within the National Tuberculosis Elimination Programme. This level is considered feasible in public health program settings while ensuring meaningful coverage and impact [15]. The M and E indicators are listed in Table 2.

**Table 2:** Indicators for implementation and effectiveness outcome proposed for CCp-K.

REAIM	
Reach	Proportion of HCW received training on CCp-K Proportion of PwTB screened for sever illness, DM, alcohol dependence and tobacco dependence Proportion of PwTB screened for sever illness, DM, alcohol dependence and tobacco dependence disaggregated by age group, gender, type of diagnostic facility
Effectiveness	Proportion of PwTB who are triage positive (overall, by specific criteria) Proportion of PwTB linked with deaddiction centre and TSC TB deaths (Early, Overall)
Adoption	Proportion of public health facilities started the practice of triage Proportion of districts that achieved indicator level targets under CCp-K Acceptance and perceived usefulness among HCW
Implementation	Proportion of triage positive patients linked to the referral centre Delay in days from diagnosis of TB to triage using CCp-K tool Facilitators and barriers for implementation Completeness of triage forms
Maintenance	No of districts included review of triage data on monthly conference Incorporation of triage tool in follow up cards Challenges to sustain the differentiated TB care model

PRISM Contextual factors	
External context	Policy Resources Guidelines Incentives
Internal context	Multi-level organizational and patient characteristics Multi-level organizational provider and patient perspectives (values) Implementation and sustainability infrastructure

**Fig. 2:**
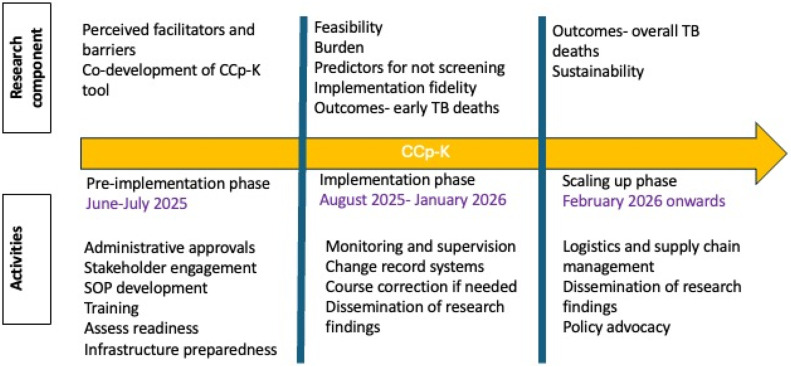
Timeline of strategies proposed in CCp-K.

The sequence of various strategies expected to be followed at different timelines are depicted in Fig. 2.

### Investigators position

In the CCp-K implementation project, the investigators play a pivotal technical and facilitative role, bridging operational delivery within Kerala's health system and evidence-based oversight. Positioned as public health experts, the investigators provide end-to-end support throughout the project lifecycle—from conceptualization and design of the comprehensive care package to implementation, monitoring, and evaluation. During the preparatory phase, they lead in developing standard operating procedures, triage tools, training materials, and referral algorithms. They guide stakeholder consultations and facilitate capacity building through training-of-trainers programs. Throughout the implementation, the investigators oversee data integrity, conduct supervisory visits, and assess fidelity through qualitative observations and periodic reviews. They also anchor the mixed-methods research component, lead the analysis of Ni-kshay and triage data, and conduct qualitative interviews to capture contextual insights. Additionally, the investigators liaise with state health authorities and WHO partners, ensuring technical alignment and advocating for policy-level integration and scale-up of the care model. Their central position ensures the scientific rigor, adaptive learning, and sustainability of this differentiated TB care intervention. The roles of Investigators, NTEP, and health system across project phases are shown in Table 3.

**Table 3:** Roles of Investigators, NTEP, and health system across CCp-K project phases.

Project Phase	Investigators	NTEP	Health System
Preparatory Phase	- Develop SOPs - Finalize triage tools - Conduct stakeholder consultations - Train trainers (ToT) - Pilot testing	- Integrate triaging into standard NTEP care processes	- Plan logistics - Prepare for human resource mobilization and facility readiness
Implementation Phase	- Provide technical oversight to NTEP - Conduct field supervision and fidelity checks - Support data systems and logistics	- Support data reporting and programmatic tracking	- Conduct triaging, referral, and documentation as routine service - Manage inpatient care
Data Collection & Monitoring	- Lead supervisory and field visits - Ensure data quality - Capture contextual insights and field notes	- Support research-related data capture and reporting systems	- Facilitate access to field sites and interviews - Provide contextual and policy-level inputs
Research & Analysis	- Lead mixed-methods design - Analyze quantitative and qualitative data - Code and synthesize interview data	- Provide support for research, review, and dissemination	- Engage decision-makers - Support policy dialogue and application of findings
Dissemination & Scale-Up	- Draft and submit reports/publications - Present findings to stakeholders and policymakers	- Coordinate with CTD and WHO for potential policy uptake	- Advocate for and operationalize scale-up within the public health system

Table 4

**Table 4:** Study variables for quantitative phase.

Objectives	List of variables	Data Source
Feasibility (coverage Timeliness Completeness) Burden	Ni-kshay ID Height (cm) Weight (kg) Swelling in leg Respiratory rate SPO2 Able to stand without support Current tobacco use Heaviness of smoking index Current alcohol use CAGE score Random Blood Sugar Fasting Blood Sugar Date of diagnosis (notification) Date of triage	Triage tool
Factors associated with non-screening & admission	Age (in completed years) Gender (Male, Female, Other) District name Smear status (Smear+/ Smear-/ Indeterminate) Site of TB (PTB/ EPTB) DST (DSTB, DRTB, Not assessed) Previous treatment status (New/ Recurrent Key population status) HIV status Treatment h/o diabetes Type of diagnostic facility type (primary / secondary / tertiary) Inpatient admission (yes/no) Type of health care providers performed the triage (Nurse, MO, MLHP, TBHV/others)	Triage tool Ni-kshay
Effect on deaths	Reported TB Deaths (within two months of treatment) Date of death	Ni-kshay

### Operational/Implementation Research (OR/ IR) component

#### Study design

This implementation project will follow an embedded mixed-methods approach. The initial qualitative phase will be followed by a quantitative phase along with descriptive qualitative phase. The quantitative phase includes secondary data extraction and qualitative phase includes in-depth interviews and content analysis of unstructured field observations. For objective 1(feasibility), 2 (burden), 3 (factors associated with not getting screened) we will use a cross-sectional study design and cohort design for objective 4 (programmatic outcomes on deaths).

### Quantitative phase

#### Study population

We will include all the patients aged 18 or more diagnosed with TB in public health facilities under NTEP in Kerala from 01 August 2025. We will include patients irrespective of their treatment initiation or transfer out status.

#### Sample size

In the 14 NTEP districts, based on the average monthly notifications in 2023, we expect around ≈20000 adult TB notifications every year from public PHIs [2]. For the OR component we expect approximately 10000 adult PwTB for six months. We shall include all patients with diagnosed TB to estimate a prevalence of ‘high risk of severe illness / triage-positive’ as low as 15% with an absolute precision of 1% at 95% confidence interval (CI). Applying the state level 8.2% death rate (2023), we expect 405 deaths in a quarter. Assuming 80% will be triaged for severe illness followed by appropriate care, we shall include 8000-triaged and 2000 non-triaged TB patients to detect a 25% reduction in death rate in triaged group when compared to non-triaged group, with an alpha error of 5% and power of 80%.

#### Data management and analysis

Source of data will be the triage forms filled by the staff nurse at PHI and Ni-kshay portal. We link the information collected from the triage form and those available with Ni-kshay using the unique “Ni-kshay ID”. We will extract the following baseline characteristics from the electronic Ni-kshay database: Ni-kshay ID, dates of notification, diagnosis, treatment initiation and treatment outcome, age, gender, test used for diagnosis, bacteriological confirmation, site of TB, previous treatment, key population status, HIV status, diabetes status, PHI type (primary / secondary / tertiary), treatment start status and transfer out (out of district) status. Treatment outcomes (early deaths and overall deaths during treatment) will be extracted after one year of date of diagnosis. Data will be analyzed using STATA ver 18 (StataCorp. 2023. Stata Statistical Software: Release 18. College Station, TX: StataCorp LLC).

### Objective wise key analytic outputs

#### Outcomes for objective 1- feasibility

To assess the feasibility, the percentage of triage coverage for comorbidity and severe illness assessment will be estimated among notified patients with TB during August 2025-January 2026. The median delay of triaging (from date of diagnosis to filling triage tool) will be summarised as median with interquartile range. The completeness of information of triaging will be summarised using percentages (%) and 95% CI (triaged for all four/three/two/one of these four conditions). Among the patients notified during August 2025 to December 2026, triaged patients accessing the care cascade will be summarised as percentages.

#### Outcomes for objective 2- burden estimates of co-morbidities

The burden of ‘high risk of severe illness / triage-positive, uncontrolled diabetes mellitus, alcohol abuse and tobacco dependence will be summarised as frequencies and %.

#### Outcomes for objective 3- predictors of not screening and not getting admitted

Patient and health system factors associated with non-triaging and not getting admitted will be described as adjusted prevalence ratio with 95% CI using log binomial regression. Not triaged for any condition and not getting admitted on triage positive will be the outcome variables and patient level and health system factors will be independent variables.

#### Outcomes for objective 4- programmatic outcomes on deaths

Early TB deaths (within 2 months of diagnosis) extracted from Ni-kshay sequentially at monthly intervals will be plotted to see trend in early TB deaths. An interrupted time series design with data on number of TB deaths will be captured for 12 months in the pre- implementation phase (August 2024 to July 2025), and five months during implementation (August 2025 to December 2025). Quarter wise number of reported TB deaths will be plotted to see trend in overall TB deaths. An interrupted time series design with data on number of TB deaths will be captured for eight quarters in the pre implementation phase (July 2023 to July 2025) and four quarters during implementation (August 2025 to August 2026). In this interrupted time series (segmented regression) model the sequentially observed absolute number of deaths will be the outcome variable. Based on the trend observed during pre-implementation and stationarity a counter factual (expected deaths without care package) will be estimated. A change level at the time of intersection between pre and post implementation and its statistical significance will be assessed. Similarly, the rate of change during the implementation adjusted for patient characteristics in subsequent quarters will be presented as co-efficient with 95% CI.

### Qualitative methods

#### Study population

We will include key stakeholders from NTEP programme and health system in the state from district and PHI level. Selected staff who are experienced, vocal and are willing to share their insights into barriers and suggest solutions will be included. Deviant case sampling of stakeholders from better performing and poor performing district will be done to understand the facilitating factors and challenges in implementing the care package. We will also refine the prototype CCp-K model with inputs from the key stakeholders. Similarly, we will include purposefully chosen TB patients (>18yrs) who underwent triaging and who did not undergo triaging to understand patient level barriers. The final list will be selected purposively (maximum variation) after brainstorming with the district NTEP stakeholders. During routine monitoring visits, the CCp-K team will also observe and discuss the challenges involved in the process of triaging and referral

#### Data collection

Barriers and suggested solutions will be explored through one-to-one in-depth interviews (IDI) by trained investigators using an interview guide. In addition, perceived suggestions for the prototype initial model developed by the research team will be captured through brainstorming. During this exercise, the proposed model will be displayed, briefed to initiate the discussion. (Annexure 2). The data will also be collected as field notes and challenges shared during field visits and the monthly review meetings. Apart from the district NTEP team, CCp-K program evaluation team also will make supervisory visits independently to capture observations from the field. This team also will hold a periodic review meeting with district program managers to discuss the field observations and program challenges.

#### Data analysis

Translation and transcription (in English) of all the IDI will be made within one week (on the same day if possible) based on the audio records and verbatim notes. The field notes, contextual interviews, informal discussions and review meeting minutes will be transcribed and coded as per the a priori defined codes identified under the PRISM frameworks related to implementation fidelity. The transcripts will be coded and thematically analyzed using open source software Taguette [31].

#### Outcomes

Through thematic analysis of in-depth interviews, field notes, and review meeting minutes, the study will identify key system-level and patient-level challenges, particularly across districts with differing performance levels. The findings will offer actionable insights into implementation fidelity, inform adaptive modifications to the triaging and referral process, and contribute to the iterative refinement of training, logistics, and monitoring strategies. Additionally, the study will capture stakeholder-driven solutions and best practices to guide scale-up and sustainability of differentiated TB care within the state health system.

### Ethics

The ethical approval is obtained from IHEC of ICMR-NIE Chennai (Approval number NIE/IHEC/A/202408-06 dated 09/08/2024). The quantitative phase will involve secondary data. For the quantitative component of the study, a verbal consent /implied consent to mimic the implementation in routine programme setting and enable assessment of feasibility subject to the approval of the ethics committee will be used. The qualitative phase will involve primary data collection. A written informed consent from the participants will be obtained before initiating the interview. As health care workers will be participating in the interview additional precautions will be taken to ensure the confidentiality of the information. We will change all identifying characteristics such as personal names, designation, district and type of facility. The location and time of interview will be selected by the participant at their convenience. The interview recordings and transcripts will be kept in password protected computers accessible only to the investigators. We will also obtain necessary administrative approvals from the Government of Kerala for implementing the CCp-K. If a patient is detected with severe TB during the implementation of CCp-K, the staff will immediately share this information with the medical officer (TU level) and/or district TB Officer for further necessary action.

### Data confidentiality

No personal identifier other than Ni-kshay ID will be captured. Electronic dataset will be kept in a password-protected computer accessible only to the investigators. Datasets and transcripts will be maintained securely for five years after completion of project.

### Specific patient and community benefits

Patients identified as severe TB, may benefit from early referral to district hospital and inpatient care. The patients diagnosed with TB may benefit in future if the strategy of triaging, referral and inpatient care of severely ill patients and those with associated co-morbid illnesses is found to be feasible and sustainable.

### Risks to participants

Although this study is conducted within the framework of routine healthcare services, potential risks to participants include discomfort during sensitive assessments (e.g., substance use), risk of stigmatization, minor logistical burden due to referrals, and risks related to data confidentiality. These are expected to be mitigated by trained staff administering assessments respectfully, ensuring private data collection, obtaining informed consent, and securely storing anonymized data. Interviews for qualitative data will be conducted at times and places chosen by participants to minimize inconvenience.

### Feedback and dissemination of results

The results of this OR will be disseminated to the Kerala state TB Office and Central TB Division. Once published in peer reviewed scientific journals, the findings will also be shared to the general public over electronic, TV, print and social media. The results will also be presented at national and international conferences and submitted for publication in a peer-reviewed journal.

### Implications for policy and practice

As this study will be conducted in routine NTEP settings, the findings will be generalizable, feasible and sustainable to be implemented on a large scale. If this strategy is found to be feasible with outcomes on reducing TB deaths, there are chances for its country wide scale up and incorporation of assessment of severe TB in routine recording and reporting. The findings of this OR will help the programme managers in first assessing the feasibility in ensuring inpatient care for all people with severe TB at notification and then prepare an operational plan to ensure its implementation including strengthening of clinical care of severe TB at district hospitals.

### Budget

The project does not require any specific funding. The quantitative phase will be implemented as a routine NTEP activity by routine health system using available workforce and resources. The qualitative component, field supervision, training material development will be supported through the NTEP/ NHM operational funds. The manuscript publication costs will be supported through the Structured Operational Research and Training Initiative (SORT IT) platform through ICMR-NIE.

## Protocol validation

Not applicable

## Limitations

Not applicable

## Declaration of competing interest

The authors declare that they have no known competing financial interests or personal relationships that could have appeared to influence the work reported in this paper.

## Data Availability

Data will be made available on request.

## Annexure A. Triaging for severe illness and comorbidities at diagnosis - Paper-based triage tool

*Ni-kshay* ID and date of notification (dd/mm/yyyy): _______________ and ______________________

NTEP ‘Diagnosis’ District: ______________ ‘Diagnosis’ TU name: ____

‘Diagnosis’ Public Peripheral Health Institution (PHI) name: ____________________

Was the TB patient admitted in hospital at the time of diagnosis/triaging (Tick one option)

ᴑ No ᴑ Yes

Weight (kilograms (kg), rounded to one decimal point - compulsory) - ___________________

Height (centimeter, no decimal point) – ___________________

BMI (kilogram per meter square (kg/m^2^), one decimal point, compulsory) - _________________

*(Use only N-TB Application for calculating BMI)*

Swelling in the leg (press for 15 sec inner side, 2 cm above the ankle), tick only one option

ᴑ No ᴑ Yes

Respiratory rate per minute (in sitting position after 30 min rest) - ___________

Oxygen saturation (%) using pulse oximeter measured after 30 min rest- ___________

Able to stand without support (tick appropriate response), choose only one option

ᴑ No (not able to stand without support) ᴑ Yes (standing without support)

High risk of severe illness / Triage-positive, tick only one option (see criterion on page 2)

ᴑ No ᴑ Yes

FBS: ___________________ mg/dl. Date of FBS (dd/mm/yyyy):

Heaviness of smoking index

1. At present, how long after waking do you wait before having your first cigarette?

1 [ ] Within 5 minutes [3 points]

2 [ ] 6-30 minutes [2 points]

3 [ ] 31-60 minutes [1 point]

4 [ ] over 60 minutes [0 points]

2. How many cigarettes per day do/did you smoke?

1 [ ] 10 or less [0 points]

2 [ ] 11-20 [1 point]

3 [ ] 21-30 [2 points]

4 [ ] 31 or more [3 points]

Nicotine dependence ᴑ No ᴑ Yes (See criterion on page 2)

CAGE criteria

1. Have you felt the need to Cut down on your drinking? ᴑ No ᴑ Yes

2. Do you feel Annoyed by people complaining about your drinking? ᴑ No ᴑ Yes

3. Do you ever feel Guilty about your drinking? ᴑ No ᴑ Yes

4. Do you ever drink an Eye-opener in the morning to relive the shakes? ᴑ No ᴑ Yes

Alcohol dependence ᴑ No ᴑ Yes (See criterion on page 2)

Date of completing triaging and filling this form at PHI (dd/mm/yyyy): _______________

Name and designation of staff completing the form: ______________________________________________________

Name, Signature (Seal) of PHI Medical Officer:

*(All details filled in this form are correct and have been cross-checked by me*)

FOR INFORMATION ONLY – Assessment criteria

If at least one of the following indicators is present (any one or more), then the adult with TB is:

‘High risk of severe illness - Yes’ / Triage-positive / Eligible for referral

If all of the following are absent, then the adult with TB is

‘High risk of severe illness - No’ / Triage-negative

Body mass index (BMI) less than or equal to (≤) 14.0 kg/m^2^

BMI 14.1 to 16.0 kg/m^2^ with leg swelling

Respiratory rate more than (>) 24 per minute

Oxygen saturation less than (<) 94%

Not able to stand without support (standing with support / squatting / sitting / bedridden)

If FBS is 250mg/dl or HbA1C>10% or more refer to nodal treatment centre for insulin and IP care

Mark nicotine dependence if score ≥4

Mark alcohol dependence Yes if 2 or more questions are answered ‘Yes’

## Annexure B. Interview guide for the stakeholder interviews

Participant ID:

Start time:

End time:

Date:

Place (be specific)/ Online:

**Introduction**

Hi, I am Dr XX. I will be interviewing you today regarding your experiences and opinions regarding differentiated TB care in Kerala state. These interviews are part of an inquiry which aims to explain the reasons for mortality among patients with TB in Kerala and develop a potential model to address these. Thank you for agreeing for the interview. Can we start?

**Question 1**

Could you please introduce yourself and your role in NTEP programme?

**Probes:**

1. Years of experience in healthcare system/health service

2. Key professional capacities

3. Experience in TB care and policy making

4. Professional capacities as a TB care professional/program/policy person

5. Specific role in NTEP and the duties currently delivered

**Question 2**

What are your thoughts on the present status of TB care and deaths due to TB in Kerala?

**Probes:**

1. Current status of TB care in the state specifically from a program perspective
2. Based on your experience as a program officer, how far have the programmatic initiatives come in the state in terms of TB care?
3. How satisfied are you with our TB related program activities?
4. What do you think of the TB related outcomes of the state in terms of recurrent infections, drug resistance and mortality?
5. What have you gathered from the stakeholders of the TB program in state regarding the activities, especially the patients and the stakeholders? What are their major complaints/reservations?
6. How do look at the mortality figures reported from Kerala compared to national figures and that of other states?

**Question 3**

In your opinion what could be the specific reasons for deaths among persons with TB in Kerala?

**Probes:**

1. Good reporting has a significant role to play with the relatively higher figures compared to other states, and we agree with that.
2. But, beyond reporting – any other gaps in programmatic initiatives contributing to high mortality?
3. Co-morbidities and their management - diabetes mellitus? The NCD program – TB program link?
4. Tobacco and alcohol?
5. Nutrition?
6. Drug availability?
7. Follow-up regularity?
8. Medication adherence?
9. Private sector?
10. Infrastructural and human resource constraints in the system?
11. Laboratories and investigations?
12. Transportation and other logistics issues?
13. Attitudes and training of the program staff?
14. Technical issues with the documentation portals
15. Financial and other constraints affecting the patients and the caregivers
16. Attitudes of the patients and the caregivers?
17. Any other

**Question 4**

Those insights are very valuable. Now based on what we know about the potential reasons for the TB related mortality in the state, how do you think we can work towards reducing them?

**Probes:**

General suggestions to address any of the programmatic gaps mentioned - strengthening the NCD program, identifying and managing diabetic patients, addressing tobacco and alcohol addiction, strengthening the implementation of nutritional support schemes like DBT, tie ups with the private sector, support for documentation, strengthening the training, better awareness among patients, efforts to address infrastructural, HR and logistics issues and so on.

Specific suggestions pointing towards a differential care model

1. Differential care for patients according to the disease severity/risk factors like nutrition, co-morbidities, and tobacco/alcohol status
2. Triaging to identify the risk status
3. Systemic efforts to focus on older patients, those with more severe symptoms/signs, co-morbidities like DM, tobacco/alcohol users, nutritional deficiencies, recurrent/drug-resistant cases and so on.
4. Any other suggestions

**Question 5**

Your suggestions point to a more targeted approach to prevention of TB-related mortality in the state by focusing on patients with severe disease or co-morbidities**.** As you maybe already aware, in 2021, the NTEP had suggested something called a differential TB care model. In this model, to reduce TB deaths in resource-limited settings, a differentiated care strategy is used to triage patients with high risk of severe illness (i.e., those with very severe undernutrition, respiratory insufficiency, or inability to stand without support) at diagnosis and refer them for comprehensive assessment and inpatient care. *The model has been successfully implemented in our neighbouring state of TN under the name of TNKET.* What do you think of such an approach for mortality reduction in Kerala?

**Probes:**

1. How relevant would such an approach be in Kerala?
2. How feasible would such an approach be?
3. What elements could be incorporated in such an approach considering the specific context of Kerala?

**Question 6**

This research team has worked out a basic design for a differential TB care model for Kerala. We would like your input to refine it for the state. Please take a look at this model. Allow the informant about 4-5 minutes to quickly go through the image. Then probe further along the following lines:

**Probes:**

1. As you see, we have conceptualized the comprehensive care pathway focusing on 4 components – disease severity, DM status, tobacco use and alcohol use. What other aspects need to be included in the pathway, if any?
2. The first component is triaging all diagnosed patients with TB using a paper-based triage tool. The triage tool includes weight, height, BMI, leg swelling, respiratory rate, Oxygen saturation, and ability to stand without support. If at least one of these is positive, the patient is eligible for referral. What are your thoughts about the triaging criteria? How valid and reliable are they? How feasible is triaging under the present circumstances? What are the barriers that you anticipate? Are there any facilitating factors?
3. The triage positive patients are referred to nodal treatment centre for assessment by physician/ pulmonologist and in patient management. This, we think could be very significant in mortality reduction. What is the current status of the nodal treatment centres? How feasible is such a referral of triage-positive patients under the present circumstances? How feasible are admissions of triage positive patients in nodal treatment centres? What infrastructural, HR and logistics gaps exist, if any, in ensuring this? What are the barriers that you anticipate? Are there any facilitating factors?
4. The second component includes the screening of all patients with TB and diabetes for fasting blood glucose (FBG) at diagnosis, and 2 months. What are your thoughts about this component? How relevant is this component for the reduction of TB-related mortality? How feasible is it to ensure that the diabetes status of all patients with TB be known to us? What is the current scenario in this regard? What infrastructural, HR and logistics gaps exist, if any, in ensuring this? What are the barriers that you anticipate? Are there any facilitating factors?
5. Further, if the FBG is ≥250 mg/dl, the patients need to be referred for insulin and inpatient care. What are your thoughts about this component? How relevant is this component for the reduction of TB-related mortality? How feasible is it to ensure that all the patients with FBG is ≥250 mg/dl receive insulin? How effective is the supply chain and storage to realise this? What is the current scenario in this regard? What infrastructural, HR and logistics gaps exist, if any, in ensuring this? What are the barriers that you anticipate? Are there any facilitating factors?
6. The third component includes the assessment of all patients with alcohol use for harmful use of alcohol and refer to deaddiction centres (“Vimukthi”) for management. What are your thoughts about this component? How relevant is this component for the reduction of TB-related mortality? How feasible is it to ensure that all the patients with alcohol use be assessed? We propose the use of CAGE questionnaire for assessment which is easy and quick. [**Describe the criteria briefly in the informant is unfamiliar with the questionnaire]**. How feasible and valid do you think the questions are? What infrastructural, HR and logistics gaps exist, if any, in ensuring this assessment and referral? What about the intake capacity and admission procedures in the Vimukthi centres? What are the barriers that you anticipate? Are there any facilitating factors?
7. The fourth and final component includes the assessment of all patients with smoking for nicotine dependence and refer to Tobacco cessation centres for moderate and high addiction. What are your thoughts about this component? How relevant is this component for the reduction of TB-related mortality? How feasible is it to ensure that all the patients with smoking be assessed for nicotine dependence? We propose the use of “Heaviness of smoking index” for assessment which is easy and quick. [**Describe the questions briefly in the informant is unfamiliar with the index]**. How feasible and valid do you think the index is? What infrastructural, HR and logistics gaps exist, if any, in ensuring this assessment and referral? What about the intake capacity and admission procedures in the tobacco cessation centres? What are the barriers that you anticipate? Are there any facilitating factors?

**Question 7**

In your opinion what are the system level facilitators and barriers for implementing a differentiated TB care model in Kerala?

**Probes:**

1. Policy level facilitators? Anything conducive for the implementation? Who could be our allies? What response do you anticipate from the health ministry? Any other government organizations? Private sector?
2. Policy level barriers if any? Competing priorities? Resource constraints at the top administrative level?
3. Who will take the initiative?
4. How about the levels of implementation? Can we start at the PHCs?
5. Human resource constraints
6. Training related constraints
7. Infrastructural constraints
8. Logistics constraints

**Question 8**

Thank you for your valuable insights. We will definitely refine the model based on your input and get back to you for your feedback.

In your opinion how can this type of a differential care model to prevent TB-related deaths be successfully implemented in the state?

**Probes:**

1. How hopeful is you about the successful implementation of this initiative?
2. Would you be willing to advocate for such a model?
3. Would you be willing to take a leadership role in its implementation?
4. Who else could we approach to discuss further about the pros and cons of this approach?
5. Who else could be our potential allies in working towards the implementation?
6. What strategies or approaches do you suggest pushing this model forward?
